# Rapid Responsiveness to Practice Predicts Longer-Term Retention of Upper Extremity Motor Skill in Non-Demented Older Adults

**DOI:** 10.3389/fnagi.2015.00214

**Published:** 2015-11-18

**Authors:** Sydney Y. Schaefer, Kevin Duff

**Affiliations:** ^1^Motor Rehabilitation and Learning Laboratory, Utah State UniversityLogan, UT, USA; ^2^Department of Physical Therapy, University of UtahSalt Lake City, UT, USA; ^3^Center on Aging, University of UtahSalt Lake City, UT, USA; ^4^Department of Neurology, University of UtahSalt Lake City, UT, USA; ^5^Center for Alzheimer’s Care, Imaging and Research, University of UtahSalt Lake City, UT, USA

**Keywords:** motor skill, learning, practice effects, retention, cognition

## Abstract

Skill acquisition is a form of motor learning that may provide key insights into the aging brain. Although previous work suggests that older adults learn novel motor tasks slower and to a lesser extent than younger adults, we have recently demonstrated no significant effect of chronological age on the rates and amounts of skill acquisition, nor on its long-term retention, in adults over the age of 65. To better understand predictors of skill acquisition in non-demented older adults, we now explore the relationship between early improvements in motor performance due to practice (i.e., rapid responsiveness) and longer-term retention of an upper extremity motor skill, and whether the extent of rapid responsiveness was associated with global cognitive status. Results showed significant improvements in motor performance within the first five (of 150) trials, and that this “rapid responsiveness” was predictive of skill retention 1 month later. Notably, the extent of rapid responsiveness was not dependent on global cognitive status, as measured by the Montreal Cognitive Assessment (MoCA). Thus, rapid responsiveness appears to be an important variable in longer-term neurorehabilitative efforts with older adults, regardless of their cognitive status.

## Introduction

The process of motor learning, as with other forms of learning, is often thought to decline with advancing age (Raz et al., 2000; King et al., 2013; Roig et al., 2014). There is, however, emerging evidence that age-related motor deficits may be more a function of peripheral factors such as sarcopenia (e.g., Macko et al., 2005) or somatosensory loss (e.g., Kalisch et al., 2008), and that certain types and metrics of motor learning are less susceptible to normal aging (Bock and Schneider, 2001; Brown et al., 2009; Bhakuni and Mutha, 2015). Importantly, much of what is known about aging and motor learning has emerged from cross-sectional studies in which data from older adults are pooled together for comparison against those from younger adults. For example, during skill acquisition, older adults tend to learn at a slower rate and to a lesser extent than younger adults (Tunney et al., 2003; Smith et al., 2005; Perrot and Bertsch, 2007; Voelcker-Rehage, 2008). Although motor learning broadly refers to improvement in the performance of sensory-guided motor behavior due to practice (Krakauer and Mazzoni, 2011), skill acquisition (or skill learning) is a specific form of motor learning that depends more on success-based exploration and multiple attempts of various movement strategies, rather than error-based adaptive processes in response to reoccurring perturbations (i.e., motor adaptation).

Studying skill acquisition in older adults may provide key insights into aging and neuroscience, as it has been shown to induce significant structural (Boyke et al., 2008; Gryga et al., 2012; Sampaio-Baptista et al., 2014) and functional (Seidler and Noll, 2008; Bezzola et al., 2011; Lauber et al., 2013; Yoo et al., 2013; Censor et al., 2014) brain changes (e.g., changes in gray matter volume, corticospinal excitability, or functional connectivity) that correlate well with measurable behavioral changes due to task practice (e.g., changes in motor sequence recall, number of movement errors, or rate of force development). In contrast to other cross-sectional studies of skill acquisition between younger vs. older adults, we have recently discovered that *within* samples of healthy older adults, chronological age itself has a minimal effect on performing and learning an upper extremity motor skill. For example, “old old” (80+ years) and “young old” (65–79 years) adults had similar degrees of motor asymmetry when performing novel unimanual tasks with their dominant vs. nondominant hands (Schaefer, 2015), and had similar rates of improvement and amounts of long-term retention after multiple days of skill training (Schaefer et al., 2015).

If one’s age is unrelated to one’s longer-term retention of motor learning (Onushko et al., 2014; Verneau et al., 2014; Schaefer et al., 2015), then how else might we predict the extent of one’s learning due to practice? Evidence from neuropsychology suggests that one’s improvements in test performance due to repeated exposures, also known as “practice effects” (Mccaffrey et al., 2000), may actually represent one’s capacity to learn over a longer-term (Duff et al., 2010). In fact, practice effects are already emerging as a key indicator of one’s cognitive status and trajectory over time (Fernandez-Ballesteros et al., 2005; Duff et al., 2011; Suchy et al., 2011; Hassenstab et al., 2015). Although such effects have been tested primarily within language and explicit memory function, they may also be applicable to procedural memory as well, which underlies the process of motor learning. Thus, the purpose of this study was to test whether practice effects in older adults predicted longer-term retention of an upper extremity motor skill, and whether the extent of any practice effect was associated with global cognitive status. Because of methodological differences between this and previous work (namely, analyzing performances from single trials collected consecutively within a session vs. performances from single testing sessions separated by days or weeks, respectively), we referred to trial-by-trial changes due to practice in this study as “rapid responsiveness” for clarity. We hypothesized that participants with more responsiveness to initial practice trials would have more skill retention 1 month later. We also hypothesized that less responsiveness to initial practice would be associated with lower cognitive status.

## Materials and Methods

### Ethics Statement

All aspects of this study were conducted in accordance with the Declaration of Helsinki, and all procedures were carried out with the adequate understanding and prior written consent of the participants as approved by Utah State University’s Institutional Review Board.

### Participants

Thirty-four right-handed adults age 65 years or older (median age: 72.5; range: 65–89) from the local community participated in this study. Twenty-four participants were female. Recruitment was based on individuals who contacted the laboratory with interest in participating as a result of approved postings throughout Cache County. Exclusion criteria included: (1) one or more self-reported neurological conditions (e.g., Parkinson’s disease, Huntington’s disease, Alzheimer’s disease, stroke, or transient ischemic attack); (2) acute or chronic musculoskeletal conditions that could affect motor function; and (3) mixed-handedness (see below).

Participants’ cognitive and sensorimotor functions were characterized in the laboratory prior to motor training. Global cognitive status was measured with the Montreal Cognitive Assessment (MoCA; Nasreddine et al., 2005), which is a reliable, easily administered, and brief cognitive screening test (max score = 30; “normal” score cutoff ≥26). General disability was recorded with the Index of Independance in Activities of Daily Living (ADL; Katz et al., 1970) in order to assess functional ability in daily life. This index is a paper-and-pencil test in which participants self-report their level of assistance needed to complete each of the six ADL functions: feeding, continence, transferring, going to toilet, dressing, and bathing. Reports of “no assistance needed” were scored as one for each ADL; thus, a score of six indicates ADL independence. We used this scale to confirm ADL independence and non-demented status regardless of age or MoCA score. Hand dominance was determined using a modified Edinburgh Handedness Questionnaire (Oldfield, 1971). Only participants with a laterality quotient of below −80% (“strongly left-handed”) or above 80% (“strongly right-handed) were included in this study. Tactile sensation was measured with Semmes Weinstein monofilaments (Touch-Test^TM^, North Coast Medical, Inc., Gilroy, CA, USA) at the distal end of the nondominant index finger on the palmar surface. We used the monofilaments sized as 2.83, 3.61, and 4.31 (manufacturer-assigned numbers that correspond to log_10_[bending force (in milligrams) × 10]; these values indicate normal touch, diminished light touch, and diminished protective sensation, respectively (Bell-Krotoski and Tomancik, 1987). Sensation was quantified for each participant as the lowest (i.e., smallest) detectable monofilament thickness. Maximal grip strength of the nondominant hand was tested via hand dynamometry (Jamar, Sammons-Preston-Rolyan, Bolingbrook, IL, USA) and measured in kilograms as the average of three consecutive measurements for each hand (Schmidt and Toews, 1970).

### Motor Skill Training

**Figure 1:**
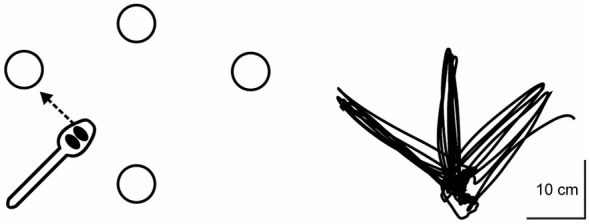
**Overhead view of motor task apparatus as well as a typical handpath over the course of one trial (i.e., five repetitions out and back to each of the three targets).** Detailed methods of kinematic data collection during the motor task are provided in Schaefer and Hengge (2015).

Participants in this study trained with their nondominant hand on a novel upper extremity motor task that we have developed previously (Schaefer et al., 2015; Schaefer, 2015). Performing this task with the nondominant hand is by design to ensure that the task is under-practiced and not over-learned, such that participants have the potential to show improvement with training (Schaefer and Lang, 2012; Schaefer, 2015). Although dominant hand performance on this task was not part of this study and its hypotheses, we have published these data previously for reference (Schaefer, 2015). Regardless of which hand is used, this task requires multijoint coordination, and has been adapted from the simulated feeding subtest of a clinical assessment that objectively measures hand function for ADL (Jebsen et al., 1969). Detailed images of the task apparatus, as well as complete instructions for task administration, have been published previously in Schaefer (2015) and Schaefer et al. (2015). To summarize, a single trial of the motor task was comprised of five repetitions to three different targets placed radially around a constant start location at a distance of 16 cm; thus, each trial equaled 15 repetitions total. The measure of performance for each trial was the time taken to complete the 15 repetitions (i.e., “trial time”), with faster times indicating better performance, as participants were instructed to “move as quickly yet as accurately as possible.” All trials were timed to the nearest 100th of a second via stopwatch. Participants were allowed to adopt any specified pattern of upper extremity kinematics during training, thereby facilitating exploratory attempts for discovering successful movement strategies for completing the task (similar to Taubert et al., 2010). Figure 1 shows an overhead view of the task apparatus as well as a typical handpath over the course of one trial (i.e., five repetitions out and back to each of the three targets). All targets were cups that were 9.5 cm in diameter and 5.8 cm in height.

Participants completed 3 days of training on the motor task in an individual setting (rather than group) within the Motor Rehabilitation and Learning Laboratory at Utah State University. All cognitive and sensorimotor tests were administered prior to motor training on day 1. We next established participants’ initial performance on the motor task based on their first trial, then proceeded to have all participants complete 50 trials per day for 3 days. Overall, the dose of skill training was 2,250 total repetitions, i.e., 15 repetitions/trial × 50 trials/day × 3 days. This dose was selected given its feasibility and efficacy for promoting skill acquisition in older adults (Schaefer et al., 2015).

To test whether early effects of practice predicted skill acquisition, we analyzed changes in performance over the first five trials from day 1. We selected this five-trial window based on our previous data (Schaefer et al., 2015) in which some participants demonstrated an asymptote in performance after only six trials, thereby indicating maximal “learning” of the task. Although most participants in that study required many more trials to achieve a behavioral asymptote, we nevertheless wanted to analyze changes in performance across trials prior to a potential plateau in performance to best test the predictability of rapid responsiveness to practice.

### Quantifying Rapid Responsiveness and Longer-Term Retention

We operationally defined “rapid responsiveness” as the normalized amount of change in motor performance from the first to fifth training trial. We computed this for each participant using the following equation (Eq. 1):

(1) $$rapid\;responsiveness\;(\%)=\frac{trial\;time_{5}-trial\;time_{1}}{trial\;time_{1}}\times 100$$

where negative values indicated improvement and the more negative the percentage, the greater the improvement. In addition to these values, we characterized rapid responsiveness by plotting trial time as a function of trial number (trial 1 through 5), and modeled the data using an exponential decay function (Eq. 2):

(2) $$y=a+be^{cx}$$

where *a* is the final trial time value that the exponential decay function approaches (i.e., asymptote), *b* is the scale of the learning from the first trial time to the value *a*, *x* is the trial number, and *c* is the rate at which learning occurs (i.e., the decay constant). This approach has been used previously to quantify upper extremity motor adaptation and learning (Martin et al., 1996; Lang and Bastian, 1999).

Because skill acquisition is often characterized as a relatively permanent change in motor performance due to practice or experience (Schmidt and Lee, 1999), we quantified the extent of skill learning based on performance on the same motor task 1 month later. We were able to re-test 24 participants (70.59%) on their nondominant hand 1 month (30–35 days) after the final day of training. At this time point, participants returned to the laboratory and performed two trials of the same upper extremity task with their nondominant hand. We then measured retention two ways: as (1) the raw average trial time (thereby accounting for performance stability and washout like Krakauer and Shadmehr, 2006; Joiner and Smith, 2008) and as (2) a percentage of each participant’s own initial performance using an equation similar to rapid responsiveness (Eq. 3):

(3) $$\text{retention}\;(\%)\text{=}\frac{\text{trial}\;{\text{time}}_{\text{one}\;\text{month}}\text{-trial}\;{\text{time}}_{\text{1}}}{\text{trial}\;{\text{time}}_{\text{1}}}\times 100$$

where the more negative the percentage, the greater the retention.

### Data and Statistical Analyses

The SAS statistical software program JMP 10.0 (SAS Institute Inc., Cary, NC, USA) was used for all statistical analysis (α=0.05). We first confirmed the presence of rapid responsiveness to practice over the first five trials using a one-way repeated-measures analysis of variance (ANOVA). When warranted by a main effect of trial number (trial 1 vs. 2 vs. 3 vs. 4 vs. 5) on trial time, Dunnett’s posthoc tests were used to determine significant differences relative to trial 1. We next fit exponential decay functions to each participants’ first five trials, and recorded the parameter estimates for *a*, which yielded a predicted asymptote in trial time. We interpreted this model estimate of trial time asymptote as the extent of skill acquisition predicted by the degree of responsiveness to a brief practice period. We then compared the predicted asymptote in trial time against the actual values of trial time at retention 1 month later using Spearman’s rank correlation coefficients (ρ). Coefficients greater than 0.59 were considered to be strong, between 0.30–0.59 were moderate, and below 0.30 were weak (Cohen, 1988).

Based on previous findings (Duff et al., 2011, 2012), we expected that the amount of rapid responsiveness and/or the retained level of performance at 1 month might have been related to global cognitive status. We therefore tested this assumption by plotting the amount of rapid responsiveness (Eq. 1) and retention (Eq. 3) against participants’ scores on the MoCA, also using Spearman’s rank correlation coefficients (ρ). Spearman’s rank correlation coefficients were used due to non-normality (Shapiro-Wilk test) or unequal variances (Welch’s test).

## Results

### Participant Characteristics

The median score on the MoCA was 25 (range: 18–30). All but one participant reported total scores of six on the Index of ADL, indicating independance on all six ADLs; the remaining one participant reported independance on all ADLs except continence, yielding a total score of 10. These data confirmed that our sample was non-demented. Of the sample, thirty-two participants were right-handed and two were left-handed based on their modified Edinburgh Handedness Questionnaire. Because they all used their nondominant hand to complete the motor task, we measured nondominant tactile sensation and grip strength prior to training for characterizing sensorimotor function. Eighteen participants had normal sensation, Thirteen had diminished light touch, and three had diminished protective sensation; none had any tactile sensory loss. Mean ± standard deviation grip strength for male participants was 36.56 ± 4.94 kg and for female participants, 19.74 ± 5.60 kg.

### Evidence of Rapid Responsiveness to Practice

**Figure 2:**
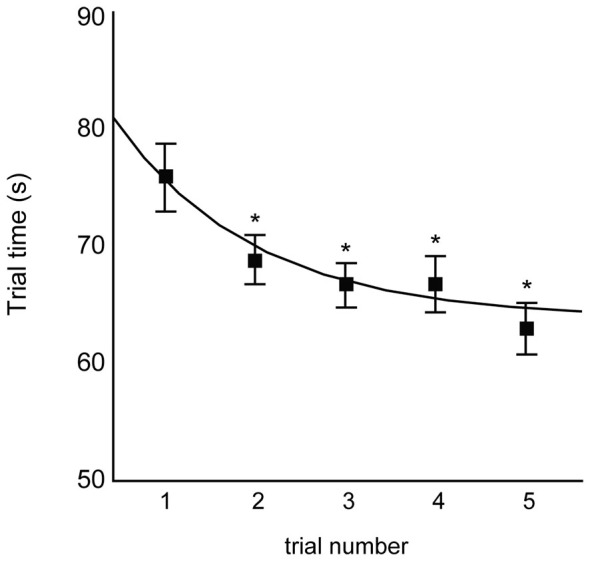
**Mean trial times for the first five trials of skill training.** Trials 2, 3, 4, and 5 were all significantly faster than trial 1 (Dunnett’s test *p < 0.05). Curve indicates exponential decay fit across all participants (*y* = 65.98 + 22.82*e*^−0.91x^).

Figure 2 illustrates the trial-by-trial improvement in motor performance, as evidenced by the decrease in mean trial time from trials 1 through 5. Repeated-measures ANOVA indicated a main effect of trial number on mean trial time (F_4,33_ = 4.82; *p* = 0.0012), with all trials having shorter trial times relative to trial 1 (all Dunnett’s tests *p* ≤ 0.05). The curve shown in Figure 2 depicts the best-fit exponential decay function across participants (see figure caption for equation). We therefore observed rapid responsiveness to practice over the first five trials of training.

### Prediction of Longer-Term Retention Based on Rapid Responsiveness to Practice

**Figure 3:**
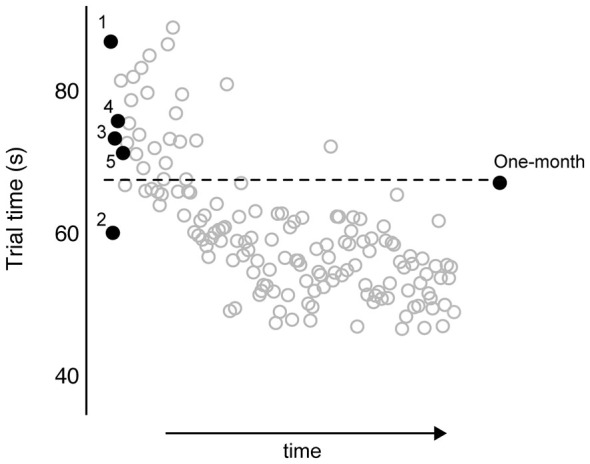
**All trial times are plotted for a representative participant.** Trial times for training trials 1–5, and at 1 month follow-up, are labeled as such and depicted as •. All remaining trials from skill training are depicted as °. Numeric value of horizontal dashed line (*y* = 63.74) was computed from exponential decay curve fitted to first five trials.

**Figure 4:**
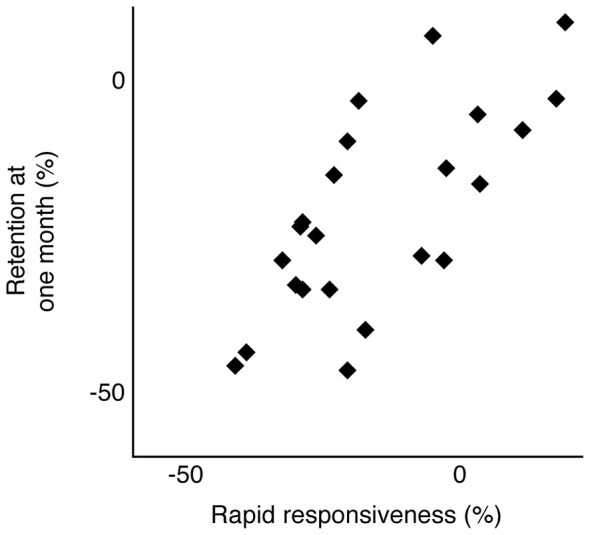
**Skill retention at 1 month is plotted as a function of rapid responsiveness across all participants available for follow-up.** Both values are expressed as a percentage of trial 1 performance (Eqs. 1 and 3), with more negative values indicating more improvement relative to trial 1. Spearman’s rank correlation coefficient, ρ = 0.66; *p* = 0.0006.

Figure 3 shows representative data from an individual participant over the course of training and at the 1 month follow-up. The trials of interest are shown in black dots and labeled as such: trials 1–5 and at 1 month. The unlabeled gray circles indicate performance from the remaining training trials. The horizontal dotted line indicates the predicted asymptote in trial time, based on the parameter estimate from the exponential decay function fitted to this participant’s data from the first five trials (*a* = 67.84). As such, this participant’s rapid responsiveness predicted the extent to which training-dependent improvements were retained 1 month later, such that this data point is close to the dotted line. Although the data in Figure 3 are from one participant, we observed a similar relationship across participants. Motor performance at 1 month following training (measured as raw average trial time) was significantly related to the predicted asymptote value across participants (Spearman’s ρ = 0.46; *p* = 0.02). To further test the relationship between rapid responsiveness and the extent of motor learning, we compared performance at 1 month against performance at trial 5, both normalized to trial 1 (Eqs. 3 and 1, respectively). Figure 4 shows the strong positive relationship between these variables ρ = 0.66; *p* = 0.0006), with large improvements by trial 5 correlating with large degrees of retention (both expressed as more negative percentages).

### No Effect of Global Cognitive Status

Although we hypothesized that less responsiveness to initial practice would be associated with lower cognitive status, we found no significant relationship between rapid responsiveness and MoCA score (relationship between these variables ρ = 0.13; *p* = 0.47). For example, in two participants who both had a MoCA score of 23, the rapid responsiveness of one was −0.64% (age 79 years) whereas the other was −32.77% (age 84 years). Similarly, MoCA score was not significantly related to trial times for trial 1 (ρ = −0.17; *p* = 0.33), trial 5 (ρ = −0.07; *p* = 0.69), or 1 month later (ρ = −0.11; *p* = 0.61) across participants.

## Discussion

The purpose of this study was to test whether practice effects in older adults predicted longer-term retention of an upper extremity motor skill, and whether the extent of any practice effect was associated with global cognitive status. Results showed that improvements in motor performance within the first five trials of practice (“rapid responsiveness”) predicted the amount of skill retention up to 1 month after training. Global cognitive status did not appear to affect the degree of rapid responsiveness. Importantly, these data replicated findings from a subsample of participants (Schaefer et al., 2015) who demonstrated and retained motor skill for 1 month, regardless of age and global cognitive status.

To our knowledge, this study is among the first to use practice effects as a means of predicting skill acquisition in older adults. Previous work in other forms of motor learning (i.e., motor adaptation) has demonstrated that young adults benefit from practice in relatively few exposure trials, and that these immediate benefits are related to the extent of adaptation after training (Joiner and Smith, 2008; Landi et al., 2011). We acknowledge that motor adaptation and skill acquisition represent different aspects of motor learning (Krakauer, 2009; Krakauer and Mazzoni, 2011), yet both may involve a rapid, early phase of learning that in a way “primes” the active neural circuitry for future consolidation occurring later in a slower, more incremental phase of learning (Karni and Sagi, 1993; Karni et al., 1998; Hauptmann et al., 2005), even in older adults (Wiggs et al., 2006). Importantly, these rapid improvements early in training have been shown to predict task performance days later in both motor adaptation and skill acquisition (Hotermans et al., 2006; Joiner and Smith, 2008; Wu et al., 2014). The brief (5–10 min) but repetitive (75 repetitions) exposure to the motor task in this study may have also induced early long-term potentiation (e.g., Frey et al., 2001), dendritic changes (e.g., Maletic-Savatic et al., 1999; Shi et al., 1999), or increased blood flow in key brain regions (e.g., Friston et al., 1992). Thus, the extent of neuroplastic changes occurring within the first five trials may be related to the extent of skill retention. Although we cannot directly test these mechanisms based our behavioral results, this study extends previous work relating rapid and longer-term improvements in motor performance to: (1) healthy older adults and (2) longer periods of retention. Given these preliminary findings, rapid responsiveness in other forms of motor learning deserves additional attention for dissociating effects of normal and abnormal aging on different neural mechanisms.

In this study, our operational definition of rapid responsiveness is consistent with the idea of practice effects within neuropsychological assessment. While practice effects are traditionally viewed as problematic when interpreting follow-up test scores, they are emerging as a means for predicting progression of cognitive decline with age (Duff et al., 2011; Hassenstab et al., 2015), presence of age-related neuropathology (Duff et al., 2014, 2015), and responsiveness to cognitive rehabilitation (Duff et al., 2010). To date, practice effects in this sense have been studied much more extensively within explicit memory function (e.g., Hopkins Verbal Learning Test—Revised, Brief Visuospatial Memory Test—Revised) and less so within implicit memory function, which plays an important role in motor learning (Carlesimo and Oscar-Berman, 1992; Milner, 2005). Interestingly, patients with Alzheimer’s disease and Mild Cognitive Impairment appear to have attenuated practice effects on explicit memory tests relative to healthy older adults (as described above), yet show comparable or even larger improvements in motor performance than healthy older adults when practicing a targeted upper extremity movement (Yan and Dick, 2006). By characterizing the relationship between early practice effects (i.e., rapid responsiveness) and retention due to motor skill training in this study, our findings further support how practice effects can probe the aging nervous system’s capacity to form multiple types of memories, be they motoric or declarative in nature.

As shown in Figure 1, participants made sizeable improvements between their first and second trials, with smaller improvements on subsequent trials. This trend in motor performance is consistent with other nonlinear changes between trials due to repeated testing on numerous cognitive assessments (Beglinger et al., 2005), even when alternate forms of each assessment were administered. Importantly, however, the first three trials in those cases were separated each by 1 week, whereas all five trials in this study were collected consecutively within the same day. Thus, the passage of time alone may not be a critical factor in understanding short-term practice effects, and also suggests that practice effects should not be considered true “learning”, which is often sensitive to periods of consolidation (Doyon et al., 2009; Inda et al., 2011). Moreover, the number of trials necessary for establishing the magnitude of practice effects is likely different for different assessments or protocols (Beglinger et al., 2005), but our exponential decay analyses suggest that the performance changes (or lack thereof) between trials 2–3, 3–4, and 4–5 are as important for determining longer-term retention as is the change from trials 1–2.

We acknowledge that this was a retrospective study by design, with all participants having completed skill training prior to the analysis of their rapid responsiveness. We also acknowledge that by recruiting participants primarily based on age (65 years and older) and lack of confounding musculoskeletal and neurological issues (see “Participants” Section), the participants reflect a convenience sample in two ways. First, 29.41% of our participants were unavailable for retention testing 1 month later for unknown reasons, thereby consistent with other attrition rates when attempting to follow older adults longitudinally over 1 month (Siddiqi et al., 2008). Second, gender was not counterbalanced within this sample, yet the inclusion of more females than males in this study is consistent with not only the typical gender distribution of adults over age 65 but also other studies of nondemented older adults (e.g., Fagan et al., 2007). Posthoc analysis nevertheless indicated that neither rapid responsiveness nor longer-term retention were unrelated to gender (both *p* > 0.79). Additionally, we acknowledge that the sole cognitive assessment used in this study (MoCA) measures only global cognition, and that the scores on this assessment ranged only from 18–30. Thus, more sensitive and specific measures of different cognitive domains may be more related to practice effects on motor learning. This study nevertheless demonstrates the potential utility of practice effects in prospectively determining who may or may not show sizeable improvements from longer-term training. Practice effects in general have already been proposed as a means for providing valuable information about diagnosis, prognosis, and treatment recommendations in memory-disordered patients with relatively little additional clinical effort or cost (Gover et al., 2014). Practice effects may also be a viable solution for improving the efficiency of clinical trials in cognitive- and physical-based rehabilitation by identifying poor responders earlier (Kraljevic et al., 2004).

## Funding

Research reported in this study was supported by the National Institute on Aging of the National Institutes of Health under award numbers K01AG047926 and R01AG045163. Additional support was provided by the Utah State University Office of Research and Graduate Studies (RC #28037) and the Marriner S. Eccles Foundation.

## Conflict of Interest Statement

The authors declare that the research was conducted in the absence of any commercial or financial relationships that could be construed as a potential conflict of interest.
